# Physical Activity Prevalence and Sex-Associated Factors Among University Students During the First Year of the COVID-19 Pandemic: A Cross-Sectional Study

**DOI:** 10.3390/sports14020070

**Published:** 2026-02-06

**Authors:** Tatiana Luz, Leonardo G. O. Luz, Bruno Giudicelli, Geraldo Neto, Liliana Baptista, Raul Martins

**Affiliations:** 1 Faculty of Sport Sciences and Physical Education, University of Coimbra, 3040-248 Coimbra, Portugallbaptista@fcdef.uc.pt (L.B.); raulmartins@fcdef.uc.pt (R.M.); 2 Proest, Federal University of Alagoas, Maceió CEP 57072-970, Alagoas, Brazil; 3 LACAPS, Federal University of Alagoas, Arapiraca CEP 57309-005, Alagoas, Brazil; bruno.giudicelli@arapiraca.ufal.br; 4 CIPER, FCDEFUC, University of Coimbra, 3040-248 Coimbra, Portugal; 5 International Clinical Research Centre (ICRC), St Anne’s University Hospital Brno (FNUSA), 602 00 Brno, Czech Republic; maranhaoneto@gmail.com

**Keywords:** health behavior, motor activity, quarantine, surveys and questionnaires young adult

## Abstract

The study investigated the prevalence of students meeting moderate-to-vigorous physical activity (MVPA) recommendations and factors associated with MVPA during the first year of the COVID-19 pandemic. A cross-sectional study was conducted with 4059 university students (2200 females [F]) during the lockdown using an electronic form. PA was assessed using the short version of the International Physical Activity Questionnaire (IPAQ-SF). Independent variables included sociodemographic characteristics, knowledge to perform PA, pre-pandemic PA, sedentary behavior (SB), and health self-reports related to the COVID-19 pandemic. Overall, 56% of students met MVPA recommendations (males [M]: 57.7%, F: 54.3%; χ^2^ = 4.703, *p* = 0.030). Knowledge to perform PA (M: OR = 3.012, 95% CI: 2.413–3.759; F: OR = 2.948, 95% CI: 2.444–3.556) and being physically active before the pandemic (M: OR = 2.651, 95% CI: 2.060–3.412; F: OR = 1.459, 95% CI: 1.079–1.974) increased the likelihood that students attained the MVPA recommendations. In contrast, longer daily exposure to SB was negatively associated with MVPA in both sexes. In this context, the present findings reinforce the relevance of universities as strategic settings for the promotion of PA in middle-income countries, where social and economic vulnerabilities may exacerbate the long-term consequences of physical inactivity.

## 1. Introduction

The health benefits of an active lifestyle are well-established in the literature [1,2]. According to the World Health Organization (WHO) guidelines on physical activity, published in 2020, to attain health benefits, adults should perform at least 150–300 min of moderate-intensity aerobic physical activity; at least 75–150 min of vigorous-intensity aerobic physical activity; or an equivalent combination of moderate- and vigorous-intensity physical activity (MVPA) during each week [3,4]. These benefits include improvements in physical and mental health, prevention and control of non-communicable chronic diseases [2] and maintenance of body weight [3,5]. Conversely, insufficient physical activity, commonly referred to as physical inactivity, is a well-established risk factor for increased morbidity and mortality, being linked to conditions such as coronary heart disease, type 2 diabetes, and cancer [6].

In 2016, the global age-standardized prevalence of insufficient physical activity was estimated at 27.5% (23.4% in men vs. 31.7% in women) [7]. This concerning situation led the WHO, in 2018, to launch the Global Action Plan on Physical Activity (GAPPA) 2018–2030, establishing a target to reduce global physical inactivity by 15% by 2030. In this context, Guthold et al. [7] estimated, for the first time, global and regional trends of insufficient physical activity based on data from 65 countries that had at least two comparable surveys. The results suggested that the global prevalence remained stable between 2001 and 2016. More recently, Strain et al. [8], analyzing data from a larger number of studies and a broader range of countries, revealed a global increase in the prevalence of physical inactivity among adults, reaching 31.3% in 2022 (28.7% in men and 33.8% in women). Women are less active than men in most countries, and both women and men become less active as they get older across all WHO regions [1].

Although research on factors associated with physical activity has increased over the past two decades, it has predominantly focused on individual-level factors (age, sex, health status, self-efficacy, and motivation) and primarily in high-income countries [9]. When considering the association between the Human Development Index (HDI) and the prevalence of physical inactivity, results revealed that less developed countries had the lowest prevalence of physical inactivity (18.7%), while physical inactivity was more prevalent among the most developed countries (27.8%) [10]. In this regard, for better understanding of the variation in the prevalence of insufficient physical activity across various contexts, the adoption of multi-domain models is essential, which provides a comprehensive perspective on the determinants of health behavior by incorporating the social and physical environments as key contributors to physical inactivity [9].

The global scenario regarding the prevalence of regular physical activity among adults in the first two decades of this millennium was already concerning [8]. However, added to this, unexpectedly, an infection caused by SARS-CoV-2, also known as COVID-19, was declared a Public Health Emergency of International Concern, and on 11 March 2020, the WHO officially declared COVID-19 a global pandemic. Several social restrictions were implemented, leading to increased sedentary behavior, reduced physical activity, disruptions in sleep patterns, changes in eating habits, and a negative impact on overall mental health, quality of life, and social interactions [11,12,13]. In Brazil, in-person classes at universities were suspended, and for a period, online classes were not conducted. These measures greatly impacted the university community, particularly due to the stressful situation of home confinement.

The university period coincides with the first years of adulthood, which makes it a critical transitional moment in which many students become susceptible to adopting unhealthy lifestyles [14]. Even before the COVID-19 pandemic, in 2019, according to the American College Health Assessment [15], fewer than 50% of college students met the minimum WHO recommendation for moderate and/or vigorous physical activity. Furthermore, a global survey conducted across 23 countries with varying income levels (low, middle, and high) revealed a physical inactivity prevalence of 41.4% among university students, with rates varying from 21.9% to 80.6% [16].

During the pandemic, several studies were published that investigated its effects on physical and mental health outcomes, as well as on quality of life among the adult population [13,17], including university students [18,19]. The impact of the lockdown on the habitual level of physical activity also emerged as a concern within the scientific community [20], prompting publications ranging from recommendations for maintaining physical activity during confinement [21,22] to studies confirming a reduction in physical activity during lockdown [23,24] in South American countries [25] and in Brazil [26]. In addition, research efforts have also sought to explore associations between physical activity and various health-related outcomes among university students, including sedentary behavior [27], eating habits [28], sleep quality [29], well-being [28] and health-related quality of life [30]. However, publications addressing correlations of physical activity within this population, particularly those that consider physical activity as the dependent variable, remain scarce, especially in the context of the COVID-19 lockdown period. This gap in the literature becomes even more pronounced when focusing on university student populations from countries in the Global South [23,24].

In view of the above, the current study aimed to investigate the prevalence of university students who met physical activity recommendations and factors associated with MVPA based on a broad set of variables from different domains during the first year of the COVID-19 pandemic.

## 2. Methods

### 2.1. Study Design and Ethical Procedures

This is an observational, cross-sectional study, with data collected via electronic questionnaire, derived from the research project entitled “*Quality of Life of Students at the Federal University of Alagoas during the COVID-19 pandemic*”. The study was approved by the Research Ethics Committee of the Federal University of Alagoas on 16 June 2020, registered under the identification CAAE 31877320.9.0000.5013. The research was carried out in accordance with the Declaration of Helsinki of 1975 [31] and complied with the Brazilian National Health Council Resolutions CNS 466/12 and CNS 510/16, which establish guidelines and regulatory standards for research involving human subjects and specific norms for studies in human and social sciences.

The informed consent form was made available for download on the first page of the electronic form, and participants were required to read and agree to its terms to proceed with the survey. To ensure anonymity, a coding system was employed, whereby each participant was identified using the initials of their full name followed by the first three digits of their Brazilian national personal identification number, as entered in the form. Participants were informed that the collected data would not allow for their personal identification and that the information would be accessed solely by the research team, provided that explicit consent was granted on the first page of the questionnaire. Furthermore, participants were guaranteed access to their own information at the conclusion of the study, upon request to the principal investigator via telephone or email, as specified in the informed consent form.

### 2.2. Participants

The undergraduate student population at the Federal University of Alagoas (UFAL), located in Alagoas, Brazil, consists of approximately 28,000 individuals, distributed across three campuses. A convenience sampling method was used. Participants were required to meet the following inclusion criteria: (a) being enrolled as an undergraduate student at UFAL, and (b) being registered on any undergraduate course in the 2020.1 academic semester. Students under the age of 18 were excluded from the study. The final sample included 4059 undergraduate students of both sexes (2200 female), enrolled in a variety of courses across the university campuses.

### 2.3. Testing Procedures and Measures

Data collection was conducted during the first year of the COVID-19 pandemic, in the period of social isolation, between June and October 2020. This period was marked by the suspension of both in-person and remote academic activities at UFAL. Due to the social distancing, the study was carried out entirely online, using an electronic form, via the Google Forms Platform, containing all the research instruments.

The survey was widely publicized on UFAL’s official website and social media. The posts included a link to the survey form. Messages with the link were also sent to students’ institutional e-mail addresses inviting them to take part in the survey. In addition, when students accessed the Academic System, information about the survey and the link to the questionnaire were displayed on the homepage. The average time required to complete the survey form was 15 min.

The dependent variable in the present study was the habitual level of physical activity, assessed using the short version of the International Physical Activity Questionnaire—IPAQ-SF [32], validated for the Brazilian adult population [33]. This instrument has been widely used in studies involving university populations [23,24,34,35]. In the IPAQ, weekly duration (min·week^−1^) is estimated for each specific physical activity, such as: (a) walking, (b) moderate-intensity physical activity (such as light cycling, swimming, dancing, light aerobics, playing recreational volleyball, carrying light weights, performing household chores around the house, yard or garden such as sweeping, vacuuming, tending the garden, or any activity that moderately increased your breathing or heart rate) and (c) vigorous-intensity physical activity (such as running, aerobics, playing football, cycling fast on a bicycle, playing basketball, doing heavy housework, yard or garden work, carrying heavy weights or any activity that greatly increased your breathing or heart rate). The combined duration of these activities represents the total weekly volume of physical activity for each participant, considering the week prior to the survey. According to the original IPAQ protocol, participants are classified into three categories based on their physical activity level: low, moderate, and high [36]. In the present study, a fourth category, ‘sedentary’, was added to classify individuals who reported no physical activity during the week. The IPAQ classification procedures can be found in Supplementary Tables S1 and S2. For analytical purposes, the sample was also dichotomized into subjects who met and did not meet the WHO recommendation for moderate-to-vigorous physical activity (MVPA) for adults [4].

The independent variables in this study were organized into domains, specifically identified as: [1] sociodemographic characteristics (sex, age, race/ethnicity, marital status, have children, place of residence, and employment status); [2] physical activity (knowledge to perform physical activity and pre-pandemic physical activity, assessed using the PAR-10 Physical Activity Rating); [3] sedentary behavior (weekly weighted average from the IPAQ-SF questions) and [4] health self-reports related to the COVID-19 pandemic. All variables are detailed in Supplementary Table S3, organized by domain, operational definitions, classification criteria within the electronic survey, and the coding schemes used for both descriptive and inferential statistical analyses.

The selection of sociodemographic variables and self-reported health indicators related to the COVID-19 pandemic was guided by prior studies examining the impact of the pandemic on adult population health [9,12,14]. As part of the PAR-10, participants were prompted to recall and report their typical physical activity habits during a regular week prior to the onset of the COVID-19 outbreak and the implementation of mobility restrictions and social distancing measures. The PAR-10 has been validated for use among Brazilian adults [37]. Pre-pandemic physical activity levels were categorized into two groups for analysis, following the classification suggested by Jurca et al. [38]: insufficiently active (inactive and low active) and active (moderately active and very active).

### 2.4. Statistical Analysis

A preliminary screening of the dataset was conducted to identify duplicate responses and verify eligibility according to the study’s inclusion and exclusion criteria. Descriptive statistics were used to characterize the sample across all study variables, both for the total sample and stratified by sex (male and female). The chi-square test was applied to examine differences in the distribution of variables according to sex.

Sex-stratified binary logistic regression models were then used to analyze the associations between independent variables and the likelihood of meeting the World Health Organization recommendations for moderate-to-vigorous physical activity (MVPA) [4]. All selected independent variables were entered simultaneously into the models to obtain adjusted odds ratios (OR) and their respective 95% confidence intervals (CI). Multicollinearity among independent variables was assessed using the variance inflation factor (VIF), with values below 5 considered acceptable. Missing data were assessed prior to analysis and handled using complete-case analysis.

Potential confounders were identified a priori based on the study’s conceptual framework and previous literature and included sociodemographic, behavioral, and COVID-19-related variables. All covariates were entered simultaneously into the sex-stratified regression models to obtain adjusted estimates.

The final analytical sample comprised 4059 participants drawn from an underlying population of approximately 25,000 university students. This sample size provides high precision for prevalence estimates and an adequate number of outcome events relative to the number of predictors included in the multivariable models, supporting stable adjusted estimates.

All statistical tests were two-sided, with a significance level set at *p* < 0.05. Statistical analyses were performed using SPSS Statistics version 28.0. Graphical representations were produced using the ggplot2 package in R software (version 4.3.3) via RStudio.

## 3. Results

Table 1 presents the descriptive characteristics of the participants summarized for the total sample and stratified by sex. The sample comprised a higher proportion of female students (54.2%), with most participants aged between 22 and 30 years (44.1%), self-identified as brown (53.2%), single (82.3%), without children (84.3%), living outside the capital (52.5%), and with no income (39.9%). Statistically significant differences were observed in the distribution of participants by sex for age group (χ^2^ = 19.678, *p* < 0.001) and employment status (χ^2^ = 62.183, *p* < 0.001).

Regarding sedentary behavior during the pandemic, the results indicated no significant differences between sexes, with 74.8% of participants reporting less than 8 h of daily sitting time.

In relation to self-reported health conditions during the COVID-19 pandemic, the university students reported not having had symptoms of COVID-19 (79.3%), been tested for COVID-19 (88.1%), a positive diagnosis (94.4%), been hospitalized due to COVID-19 (60.3%), made contact with individuals who tested positive for COVID-19 (60.3%), a history of chronic illness or obesity (81.6%), and access to private health insurance (76.8%). Furthermore, most students reported complying with social isolation guidelines, leaving home only for essential reasons (66.2%), sleeping between 7 and 9 h per night (60.7%), experiencing weight gain during the pandemic period (50.5%) and not participating in any extracurricular academic activities while in isolation (55.8%). Sex-based comparisons revealed that female students, compared to male students, were less commonly tested for COVID-19 (M: 86.2%; F: 89.8%; χ^2^ = 12.112, *p* < 0.001), adhered more to social isolation guidelines (M: 75.3%; F: 98.4%; χ^2^ = 92.069, *p* < 0.001) and had a higher prevalence in the group that reported sleeping at least 9 h a day (M: 15.8%; F: 23.0%; χ^2^ = 33.503, *p* < 0.001).

**Figure 1:**
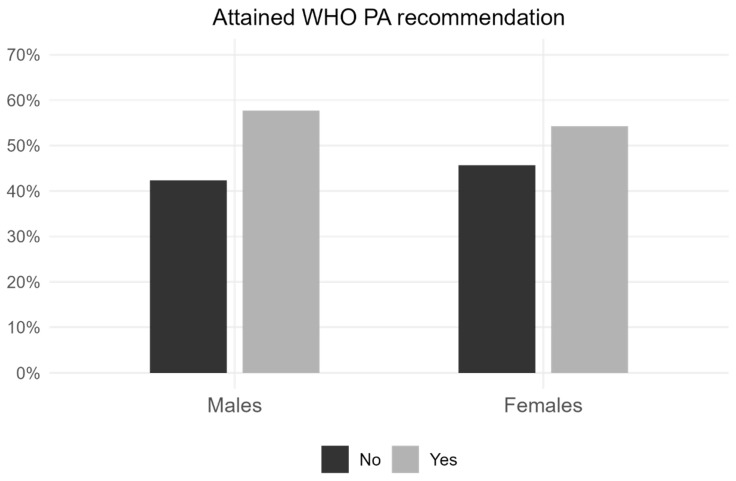
Prevalence of university students who meet and do not meet the WHO recommendations for MVPA, according to gender: male (n = 1859) and female (n = 2200). Chi-squared test: χ^2^ = 4.703, *p* = 0.030. MVPA = moderate-to-vigorous physical activity; PA = physical activity; WHO = World Health Organization.

The findings related to physical activity indicate that most students demonstrated a satisfactory level of physical activity during the pandemic, according to both the IPAQ classification (57.1%) and the WHO MVPA recommendation (55.8%). Furthermore, significant differences between sexes were revealed. Male students (57.7%) were slightly more active than female students (54.3%; χ^2^ = 4.703, *p* = 0.030) (Figure 1), and had greater engagement in vigorous physical activity (31.4%), whereas female students practiced more moderate physical activity (30.2%; χ^2^ = 16.154, *p* < 0.001). Additionally, males reported having more knowledge to perform physical activity than females (M: 70.9%; F: 64.3%; χ^2^ = 37.147, *p* < 0.001) and were also more physically active before the pandemic (M: 25.8% active; F: 10.8% active; χ^2^ = 155.750, *p* < 0.001).

As presented in Table 2, the results of the binary logistic regression models, analyzed separately for male and female students, indicate that, regardless of sex, both knowledge to perform physical activity (M: Odds Ratio [OR] = 3.012, 95% Confidence Interval [CI]: 2.413–3.759; F: OR = 2.948, 95% CI: 2.444–3.556) and being physically active before the pandemic (M: OR = 2.651, 95% CI: 2.060–3.412; F: OR = 1.459, 95% CI: 1.079–1.974) increased the likelihood that students attained the MVPA recommendations of the WHO during the pandemic. In contrast, longer daily exposure to sedentary behavior was negatively associated with MVPA (M: OR = 0.558, 95% CI: 0.444–0.702; F: OR = 0.501, 95% CI: 0.406–0.618). Among male students, having pre-existing chronic diseases or obesity (OR = 0.760, 95% CI: 0.582–0.992), and sleeping between 7 and 9 h per night (OR = 1.327, 95% CI: 1.029–1.713), were also significantly associated with MVPA recommendations. For female students, leaving the house a few times in special situations, during the period of social isolation, contributed to an increase likelihood of meeting the WHO’s MVPA guidelines in the first year of the pandemic (OR = 1.676, 95% CI: 1.261–2.226).

## 4. Discussion

The findings of the present study indicated that more than half of the university students met the WHO recommendations for moderate-to-vigorous physical activity (MVPA) during the first year of the COVID-19 pandemic. Males showed a slightly higher prevalence of sufficiently active individuals compared to females. Among the factors associated with MVPA engagement during the period of social isolation, prior knowledge to perform physical activity and the adoption of an active lifestyle before the COVID-19 pandemic stood out as important predictors in both sexes. Conversely, greater daily exposure to sedentary behavior appeared to negatively impact sufficient participation in MVPA.

Prior to the COVID-19 pandemic, the global prevalence of adults engaging in sufficient physical activity was approximately 72.5% [1,7]. In Brazil, data from the latest pre-pandemic *Surveillance of Risk and Protective Factors for Chronic Diseases by Telephone Survey in Brazil (VIGITEL)*, collected in 2019 [39], indicated a prevalence of 56.1% of physically active adults, reaching 62.7% among individuals with 12 or more years of education. Among university students, a study involving 24 low- and middle-income countries reported that 57.7% were considered physically active [40]. In the present study, based on participants’ self-reports, the prevalence of physically active university students before the pandemic was 17.7%, a value significantly lower than both global and national estimates, including that of the Brazilian population with 12 or more years of education.

Based on the data collected using the IPAQ-SF, during the COVID-19 lockdown, this study found a prevalence of 57.1% of physically active university students. In the same year, data from the 2020 VIGITEL survey [39], which employed a similar questionnaire, reported a prevalence of 59.0% of physically active adults with 12 or more years of education. Notably, the literature presents mixed findings on the impact of the pandemic on physical activity levels among university students. Data from the American College Health Assessment [15,41] indicate an increase in the proportion of university students meeting the WHO guidelines for MVPA, rising from 46.2% in 2019 to 64.5% in 2020. Similarly, Romero-Blanco et al. [42], in a study involving health sciences students, reported an increase in physical activity levels during the lockdown period. In contrast, some studies have shown opposite results, with a reduction in physical activity during lockdown in university students across several countries [23,24,43,44] and in students and university staff from various regions of Brazil [27]. In Brazil during the pandemic, Martins et al. [26] reported a decline of more than 15% in physical activity practice from pre to during the pandemic, and for university students following the lockdown protocols of staying at home, this percentage was even lower for leisure-time physical activity. These results suggest that during the COVID-19 pandemic, there were different findings across the globe that could be due to different methodological study designs, different instruments to assess physical activity (self-reported versus objectively measured), different cultural backgrounds (South America versus European), distinct participant sociodemographic characteristics, and the complex interplay of individual and contextual factors (e.g., employment, mental health status, residency location) [9], which may help to explain the low prevalence observed among university students. In addition, it is important to note that the instrument used in this study to assess habitual physical activity prior to the pandemic was the PAR-10, which limits the evaluation to the leisure-time domain. This characteristic differentiates it from the instruments used in the aforementioned surveys, which typically assess multiple domains of physical activity, including occupational, transportation-related, and household activities [1,7,39].

Concerning sex-related differences, there are also inconsistent findings in the current literature. In a study conducted among university students in Southeast Asia during the pandemic, authors reported a higher proportion of female students (54.3%) that met the recommended levels of physical activity compared to their male counterparts (45.7%) [45]. Similar findings were reported by Goicochea et al. [43], who attributed the difference to a more pronounced decline in physical activity levels among male students during the pandemic. Supporting this interpretation, a systematic review by López-Valenciano et al. [23] reported a more pronounced reduction in physical activity among men compared to women. As observed in the current study, the data revealed lower adherence to social isolation measures by men (75.3%) compared to women (98.4%), potentially contributing to the higher male prevalence of physically active individuals, confirming the trend previously reported in the pre-pandemic literature. Regarding the sex differences, the current study also found a higher male prevalence compared to females (25.8% versus 10.8%, respectively), which is consistent with the literature, which shows higher levels of engagement in MVPA among males, regardless of age group, measurement instrument, or context analyzed [1,7,39].

Recent studies continued to examine the physical activity behaviors of university students during the COVID-19 pandemic [27,46,47] on distinct health outcomes. The sustained academic interest in this topic reflects the unique opportunity to investigate behavioral patterns under conditions of social restriction, a scenario imposed by the pandemic but already emerging progressively in the years preceding it. This shift has been closely linked to a decline in university students’ participation in physical activity, with adverse effects on various health-related indicators, including well-being, quality of life, and psychological symptoms such as depression, anxiety, and stress, irrespective of age [48]. For instance, in a recent systematic review, Martins et al. [49] showed that the COVID-19 pandemic had a negative impact on the mental health of university students related to symptoms of anxiety, depression, and stress, most of them associated with poorer sleep quality and emotional changes due to the reduction in physical activity patterns. A systematic review by López-Valenciano et al. [23] reported that students who were sufficiently active prior to confinement tended to remain active during the lockdown, despite an overall decline in physical activity levels. Similar results were reported by Gallè and colleagues [44], who found that 44.7% of university students remained physically active during the pandemic, with previous engagement in physical activity being positively associated with compliance with recommended activity levels. These findings highlight the importance of pre-pandemic knowledge to perform physical activity and the maintenance of an active lifestyle as key predictors of adherence to physical activity guidelines during the lockdown, regardless of sex, which is further supported by the findings of our study.

Another behavioral change associated with the period of social isolation was the increase in sedentary behavior across various populations [50], including university students [24,44,51]. As observed in the current study, daily time spent in sedentary behaviors was found to be negatively associated with physical activity, suggesting that reducing sedentary time may represent an effective strategy for promoting physical activity among both male and female students. Amornsriwatanakul et al. [45] reported that university students who spent more than 8 h/day on sedentary behavior were 32% less likely to meet MVPA recommendations compared to those who spent ≤3 h/day on sedentary activities. Several additional variables were also associated with physical activity levels during the pandemic, including social isolation [46], fear related to the pandemic [46], the presence of chronic diseases [47], negative health perception [47], and depressive symptoms [48]. In the current investigation, among male students, the presence of chronic conditions or obesity, as well as the habit of sleeping between 7 and 9 h/night, were positively associated with sufficient physical activity. On the other hand, among female students, being able to leave the house sometimes was positively associated with meeting physical activity recommendations. These findings suggest different sex-related factors to maintain physical activity recommendations. In addition, these findings underline that interventions aimed at increasing knowledge to perform physical activity, along with efforts to reduce sedentary time, may be effective in improving the health and well-being of university students of both sexes, particularly in contexts of social restriction. Therefore, while the pandemic presented challenges (social, mental, and physical), it offered opportunities to reinforce the importance of well-being, mental health and support systems within the academic environments [48,52] through the increase in healthy physical activity patterns while reducing sedentary behaviors. Interventions targeted to increase physical literacy among university students, as well as physical activity interventions (e.g., online), may serve to protect against future pandemic crises.

The current study presents several strengths, including the collection of data during the first year of the COVID-19 pandemic, a large sample size, the inclusion of a comprehensive set of independent variables in the analytical model to better understand the physical activity behavior of university students, and the use of validated instruments for assessing physical activity in the Brazilian adult population. Despite these methodological strengths, some limitations should be acknowledged: (a) the cross-sectional design does not allow for the establishment of causal relationships between the variables studied; (b) the use of a Brazilian convenience sampling method may limit the generalizability of the findings to other global populations given the sociocultural characteristics of the university students in Brazil as well as the restrictions measures adopted during the COVID-19 lockdown in Brazil; (c) data were obtained through self-reported questionnaires, which may increase the risk of misinterpretation of the items as well as recall bias regarding the type, frequency, and duration of physical activity; (d) the instrument used to assess habitual physical activity levels in the pre-pandemic period (PAR-10) restricts analysis to the domain of leisure-time activity, excluding other relevant domains such as commuting, work, and household activities; (e) although the IPAQ-SF is a validated tool, it does not provide a comprehensive understanding of the context in which the physical activity is performed, despite accounting for different types and intensities of activity; the use of more objective measures are recommend for future studies in this topic; (f) we cannot exclude the influence of other non-measured confounding variables such as additional employment, local of residency, mental health status and online resources to physical activity. In addition, data collection occurred between June and October 2020, a period characterized by evolving phases of the COVID-19 pandemic in Brazil and varying levels of public health restrictions; therefore, participants may have responded under different contextual conditions, which could have influenced physical activity behaviors and introduced potential temporal bias.

The study identified the relevance of knowledge about physical activity, prior experience in these activities, and less time spent in sedentary habits as factors associated with maintaining adequate levels of physical activity during the COVID-19 pandemic. The results reinforce the need to expand health education actions focused on disseminating evidence-based information about the benefits and guidelines for physical activity in order to promote engagement even in contexts of social restrictions. Prior experience with physical activity emerged as a significant predictor of adherence and persistence in MVPA recommendations during adverse situations, highlighting the importance of implementing programs to encourage regular practice from childhood to consolidate healthy lifestyle habits. Interventions should take into account the heterogeneity of the population, respecting functional limitations, individual preferences, and socioeconomic contexts. Finally, the results of the present study reinforce the need for comprehensive and sustained approaches, anchored in knowledge and practice of physical activity, as central elements for promoting the health [28,29,53] and quality of life [30] of university students, especially during global health crises such as the COVID-19 pandemic.

## 5. Conclusions

The findings of the present study indicate that, although the lockdown imposed substantial challenges to engagement in physical activity among Brazilian university students, more than half of the participants still achieved the WHO recommendations for MVPA during the first year of the COVID-19 pandemic. The results suggest that individual-level characteristics, such as knowledge on how to perform physical activity and pre-pandemic physical activity habits, together with behavioral and contextual factors, including sedentary time and opportunities to leave home, were statistically associated with meeting these recommendations in both sexes. The knowledge to perform physical activity emerges as a key element in fostering individual autonomy to adopt and maintain an active lifestyle, regardless of the sociodemographic factors involved.

In this context, the present findings reinforce the relevance of universities as strategic settings for the promotion of physical activity in middle-income countries, where social and economic vulnerabilities may exacerbate the long-term consequences of physical inactivity. These implications should be interpreted in light of the study’s cross-sectional design and reliance on self-reported measures. Integrating actions to promote physical activity into university curricula and institutional policies, such as providing structured opportunities to develop practical skills for engaging in physical activity, implementing measures to reduce sedentary time, and creating or improving safe and accessible environments for movement within and around the campus, may contribute to the adoption and maintenance of more active lifestyles among students of both sexes. Furthermore, longitudinal and intervention studies conducted in the post-pandemic period and in different higher education contexts in the Global South are warranted to monitor changes in physical activity patterns over time, to clarify temporal relationships between ecological determinants and physical activity, and to test multilevel strategies aimed at strengthening knowledge, autonomy, and supportive environments for physical activity.

## Figures and Tables

**Table 1:** Descriptive statistics for categorical variables in university students and results of the chi-squared test between groups stratified by sex (n = 4059).

Domains/ Variables	Descriptive Statistics						Chi-Squared	
	Total (n = 4059)		Males (n = 1859)		Females (n = 2200)		χ^2^	*p*
	n	(%)	n	(%)	n	(%)		
**Sociodemographic characteristics**								
*Age (years)*								
≤21	1580	(38.9)	691	(37.2)	889	(40.4)		
22–30	1790	(44.1)	800	(43.0)	990	(45.0)		
>30	689	(17.0)	368	(19.8)	321	(14.6)	19.678	<0.001
*Race or ethnicity*								
Asian (yellow)	113	(2.8)	45	(2.2)	68	(3.1)		
White	1153	(28.4)	524	(28.2)	629	(28.6)		
Village-dwelling indigenous	5	(0.1)	2	(0.1)	3	(0.1)		
Indigenous	17	(0.4)	6	(0.3)	11	(0.5)		
Brown	2156	(53.2)	1003	(54.0)	1153	(52.4)		
Black not-quilombola	459	(11.3)	221	(11.9)	238	(10.8)		
Black quilombola	26	(0.6)	10	(0.5)	16	(0.7)		
NR	130	(3.2)	48	(2.6)	82	(3.7)	8.670	0.277
*Marital status*								
Single	3341	(82.3)	1516	(81.5)	1825	(83.0)		
Married/Domestic partnership	616	(15.2)	304	(16.4)	312	(14.2)		
Separated/Divorced	95	(2.3)	37	(2.0)	58	(2.6)		
Widowed	7	(0.2)	2	(0.1)	5	(0.2)	6.005	0.111
*Have children*								
No	3421	(84.3)	1556	(83.7)	1865	(84.8)		
Yes. 1 or 2	551	(13.6)	258	(13.9)	293	(13.3)		
Yes. 3 or more	87	(2.1)	45	(2.4)	42	(1.9)	1.601	0.449
*Place of residence*								
Non-capital	2129	(52.5)	945	(50.8)	1184	(53.8)		
Capital	1930	(47.5)	914	(49.2)	1016	(46.2)	3.598	0.058
*Employment status*								
Retired	20	(0.5)	15	(0.8)	5	(0.2)		
Self-employed	193	(4.8)	96	(5.2)	97	(4.4)		
Unemployed	548	(13.5)	248	(13.3)	300	(13.6)		
Scholarship student	806	(19.9)	318	(17.1)	488	(22.2)		
Intern student	162	(4.0)	85	(4.6)	77	(3.5)		
Unpaid student	1620	(39.9)	698	(37.5)	922	(41.9)		
Private sector employee	434	(10.7)	234	(12.6)	200	(9.1)		
Public servant	276	(6.8)	165	(8.9)	111	(5.0)	62.183	<0.001
**Sedentary behavior**								
*Sitting time (hours)*								
<8 h	3037	(74.8)	1368	(73.6)	1669	(75.9)		
≥8 h	1022	(25.2)	491	(26.4)	531	(24.1)	2.770	0.096
**Health self-reports related to the COVID-19 pandemic**								
*Symptoms of COVID-19*								
No	3217	(79.3)	1486	(79.9)	1731	(78.7)		
Yes	842	(20.7)	373	(20.1)	469	(21.3)	0.963	0.326
*Tested for COVID-19*								
No	3578	(88.1)	1603	(86.2)	1975	(89.8)		
Yes	481	(11.9)	256	(13.8)	225	(10.2)	12.112	<0.001
*Diagnosed with COVID-19*								
No	3832	(94.4)	1741	(93.7)	2091	(95.0)		
Yes	227	(5.6)	118	(6.3)	109	(5.0)	3.703	0.054
*Hospitalization due to COVID-19*								
No	4026	(99.2)	1849	(99.4)	2177	(99.0)		
Yes	33	(0.8)	10	(0.5)	23	(1.0)	3.219	0.073
*Contact with someone diagnosed with COVID-19*								
No	2446	(60.3)	1109	(59.7)	1337	(60.8)		
Yes	1613	(39.7)	750	(40.3)	863	(39.2)	0.525	0.469
*Chronic diseases or obesity*								
No	3311	(81.6)	1537	(82.7)	1774	(80.6)		
Yes	748	(18.4)	322	(17.3)	426	(19.4)	2.796	0.094
*Health insurance*								
No	3119	(76.8)	1401	(75.4)	1718	(78.1)		
Yes	940	(23.2)	458	(24.6)	482	(21.9)	4.213	0.040
*Behavior to prevent the spread of COVID-19*								
Total social isolation	478	(11.8)	227	(12.2)	251	(11.4)		
Leave few times	2687	(66.2)	1103	(59.3)	1584	(72.0)		
Leave to work	789	(19.4)	70	(3.8)	330	(15.0)		
Not in social isolation	105	(2.6)	459	(24.7)	35	(1.6)	92.069	<0.001
*Sleep time (hours)*								
<7 h	794	(19.6)	391	(21.0)	403	(18.3)		
7 h–9 h	2465	(60.7)	1174	(63.2)	1291	(58.7)		
>9 h	800	(19.7)	294	(15.8)	506	(23.0)	33.503	<0.001
*Body weight changed*								
Decreased	803	(19.8)	346	(18.6)	457	(20.8)		
Remained the same	1206	(29.7)	629	(33.8)	577	(26.2)		
Increased	2050	(50.5)	884	(47.6)	1166	(53.0)	27.927	<0.001
*Extra (others) academic activities*								
No	2264	(55.8)	1084	(58.3)	1180	(53.6)		
Yes	1795	(44.2)	775	(41.7)	1020	(46.4)	8.926	0.003
**Physical Activity**								
*IPAQ classification*								
Sedentary	804	(19.8)	358	(19.3)	446	(20.3)		
Low intensity	937	(23.1)	416	(22.4)	521	(23.7)		
Moderate intensity	1166	(28.7)	501	(26.9)	665	(30.2)		
High intensity	1152	(28.4)	584	(31.4)	568	(25.8)	16.154	0.001
Attained WHO PA recommendation *								
No	1793	(44.2)	787	(42.3)	1006	(45.7)		
Yes	2266	(55.8)	1072	(57.7)	1194	(54.3)	4.703	0.030
*Knowledge to perform PA*								
None	331	(8.2)	131	(7.0)	200	(9.1)		
Poor	995	(24.5)	410	(22.1)	585	(26.6)		
Good	2329	(57.3)	1085	(58.4)	1244	(56.5)		
Very good	404	(10.0)	233	(12.5)	171	(7.8)	37.147	<0.001
*PA habits before COVID-19 pandemic*								
Insufficiently active	3341	(82.3)	1379	(74.2)	1962	(89.2)		
Active	718	(17.7)	480	(25.8)	238	(10.8)	155.750	<0.001

Abbreviations: IPAQ = International Physical Activity Questionnaire; NR = not reported; PA = physical activity; WHO = World Health Organization. * WHO PA recommendation (2020) [4].

**Table 2:** Logistic regression model estimating the Odds-Ratio for attained WHO Physical Activity (PA) recommendation in males and females.

Independent Variables	Attained WHO PA Recommendation			
	Males ^a^ (n = 1859)		Females ^b^ (n = 2200)	
	Odds Ratio	95% CI	Odds Ratio	95% CI
**Sociodemographic characteristics**				
*Age (years)*				
≤21 *	1		1	
22–30	1.105	0.874–1.397	0.889	0.727–1.087
>30	0.918	0.636–1.324	0.928	0.662–1.301
*Race or ethnicity*				
White/Yellow/Indigenous *	1		1	
Brown/Black	1.225	0.985–1.523	0.868	0.716–1.052
NR	1.469	0.743–2.904	0.933	0.573–1.519
*Marital status*				
Single/Separated/Divorced/Widowed *	1		1	
Married/Domestic partnership	0.845	0.579–1.235	1.067	0.803–1.420
*Have children*				
No *	1		1	
Yes	1.154	0.774–1.719	0.947	0.690–1.299
*Place of residence*				
Non-capital *	1		1	
Capital	0.899	0.734–1.102	0.880	0.735–1.054
*Employment status*				
Student with no income *	1		1	
Student with income	0.821	0.655–1.028	0.924	0.761–1.122
**Sedentary behavior**				
*Sitting time (hours)*				
<8 h *	1		1	
≥8 h	0.558	0.444–0.702	0.501	0.406–0.618
**Health self-report related COVID-19 pandemic**				
*Symptoms of COVID-19*				
No *	1		1	
Yes	0.854	0.659–1.108	0.984	0.783–1.236
*Contact with someone diagnosed with COVID-19*				
No *	1		1	
Yes	0.943	0.757–1.174	1.002	0.824–1.218
*Chronic disease or obesity*				
No *	1		1	
Yes	0.760	0.582–0.992	1.018	0.811–1.278
*Behavior to prevent the spread of COVID-19*				
Total social isolation *	1		1	
Leave few times	1.005	0.734–1.375	1.676	1.261–2.226
Leave to work/Not in social isolation	1.157	0.803–1.668	1.314	0.919–1.880
*Sleep time (hours)*				
<7 h *	1		1	
7 h–9 h	1.327	1.029–1.713	1.013	0.797–1.288
>9 h	0.825	0.590–1.153	0.871	0.657–1.155
**Physical Activity**				
*Knowledge to perform PA*				
None/Poor *	1		1	
Good/Very good	3.012	2.413–3.759	2.948	2.444–3.556
*PA habits before COVID-19 pandemic*				
Insufficiently active *	1		1	
Active	2.651	2.060–3.412	1.459	1.079–1.974

Abbreviations: NR = not reported; PA = physical activity; WHO = World Health Organization.; ^a^ The logistic regression model explained 19.2% (Nagelkerke *R*^2^) of the variance in the performance in males (Omnibus test: χ^2^ (18) = 285.944; *p* < 0.001 and Hosmer and Lemeshow test: χ^2^ (8) = 3.271; *p* = 0.916). ^b^ The logistic regression model explained 13.1% (Nagelkerke *R*^2^) of the variance in the performance in females (Omnibus test: χ^2^ (18) = 226.207; *p* < 0.001 and Hosmer and Lemeshow test: χ^2^ (8) = 13.537; *p* = 0.095). * Reference category.

## Data Availability

The data presented in this study are available on request from the corresponding author.

## Supplementary Materials

The following supporting information can be downloaded at: https://www.mdpi.com/article/10.3390/sports14020070/s1, Table S1: Formula for calculating IPAQ-SF continuous scores expressed as MET-minutes per week; Table S2: International Physical Activity Questionnaire—IPAQ-SF (short form) categorical score; Table S3: Domains, characterization, categorization, and coding of the study variables for descriptive and inferential analyses.
