# Supplementary material for: Physical Activity Prevalence and Sex-Associated Factors Among University Students During the First Year of the COVID-19 Pandemic: A Cross-Sectional Study
Source: Sports (Basel). 2026 Feb 6;14(2):70. doi: 10.3390/sports14020070 (PMC12944473; doi:10.3390/sports14020070)
Supplement: Supplementary file 1 [file sports-14-00070-s001.zip › sports-3993402-supplementary.pdf]

Supplementary table 1. Formula for calculating IPAQ-SF continuous scores expressed as MET-minutes per week.

PA Level (MET-Minutes/Week)	Calculation Formulas
High intensity	8.0 x minutes of high intensity activity/day x days per week
Moderate intensity	4.0 x minutes of moderate activity/day x days per week
Low intensity	3.3 x minutes of walking/day x days per week

Abbreviations: IPAQ-SF = International Physical Activity Questionnaire - short form; MET = metabolic equivalent task; PA = physical activity

Supplementary table 2. International Physical Activity Questionnaire – IPAQ-SF (short form) categorical score.

**PA Levels**	**Category Criteria**
High intensity	• high-intensity PA on at least 3 days and accumulating at least 1500 MET-min/week, OR<br>• 7 days or more of any combination of walking, moderate- or high-intensity activities accumulating at least 3000 MET-min/week
Moderate intensity	• 3 or more days of high activity of at least 20 minutes per day, OR<br>• 5 or more days of moderate-intensity activity and/or walking of at least 30 minutes per day, OR<br>• 5 or more days of any combination of walking, moderate-intensity or high intensity activities achieving a minimum of at least 600 MET-min/week
Low intensity	• some activity is reported, but not enough to meet the above criteria for moderate- and high-groups level
Sedentary	• no activity is reported

Abbreviations: IPAQ-SF = International Physical Activity Questionnaire - short form; MET = metabolic equivalent task; PA = physical activity
PA (Physical activity).

Supplementary table 3. Domains, characterization, categorization, and coding of the study variables for descriptive and inferential analyses.

Domains	Characterization of the variable	Variable according to the electronic form	Categorization in the electronic form	Categorization for analysis	Coding for descriptive analysis and Chi-square	Coding for logistic regression analysis
Sociodemographic characteristics	Sex	Sex	Females	Females	0	-
			Males	Males	1	-
	Age	Age (years)	Open-ended question	≤ 21 years	0	0
				22 – 30 years	1	1
				> 30 years	2	2
	Race or ethnicity	What is your race or ethnicity?	Asian (yellow)	Yellow	0	0
			White	White	1	
			Village-dwelling indigenous	Village-dwelling indigenous	2	
			Indigenous	Indigenous	3	
			Brown	Brown	4	1
			Black not-quilombola	Black not-quilombola	5	
			Black quilombola	Black quilombola	6	
			Not reported	NR	7	2
	Marital status	Marital status	Single	Single	0	0
			Separated / Divorced	Separated / Divorced	2	
			Widowed	Widowed	3	
			Married / Domestic partnership	Married / Domestic partnership	1	1
	Have children	Do you have children?	No	No	0	0
			Yes. 1 or 2	Yes. 1 or 2	1	1
			Yes. 3 or more	Yes. 3 or more	2	
	Place of residence	In which city do you live?	Open-ended question	Non-capital	0	0
				Capital	1	1
	Employment status	What is your employment status?	Unemployed	Unemployed	2	0
			Unpaid student	Unpaid student	5	
			Retired	Retired	0	1
			Self-employed	Self-employed	1	
			Scholarship student	Scholarship student	3	
			Intern student	Intern student	4	
			Private sector employee	Private sector employee	6	
			Public servant	Public servant	7	
Physical Activity	IPAQ classification	IPAQ-SF	IPAQ-SF	Sedentary	0	-
				Low intensity	1	-
				Moderate intensity	2	-
				High intensity	3	-
	Attained WHO PA recommendation*	IPAQ-SF	IPAQ-SF	No	0	0
				Yes	1	1
	Knowledge to perform PA	How do you assess your knowledge to perform physical activities? (knowing what to do and how to do it)	I have no knowledge at all	None	0	0
			Poor	Poor	1	
			Good	Good	2	1

			Very good	Very good	3	
	PA habits before Covid-19 pandemic	PAR-10	PAR-10	Insufficiently active	0	0
				Active	1	1
Sedentary behavior	Sitting time	How much time do you spend sitting in total during a weekday? How much time do you spend sitting in total during a weekend day?	Open-ended question (Calculation of the weighted average)	< 8h	0	0
				≥ 8h	1	1
Health self-reports related Covid-19 pandemic	Since the beginning of the Covid-19 pandemic...	Have you had or are you currently experiencing symptoms of Covid-19?	No	No	0	0
			Yes	Yes	1	1
		Have you been tested for Covid-19	No	No	0	0
			Yes	Yes	1	1
		Have you been diagnosed with Covid-19?	No	No	0	0
			Yes	Yes	1	1
		Did you require hospitalization due to Covid-19?	No	No	0	0
			Yes	Yes	1	1
		Have you been in contact with someone diagnosed with Covid-19?	No	No	0	0
			Yes	Yes	1	1
		Do you have any pre-existing chronic diseases or obesity?	No	No	0	0
			Yes	Yes	1	1
		Do you have health insurance?	No	No	0	0
			Yes	Yes	1	1
		How is your behavior in trying to prevent the spread of Covid-19?	I have been in total social isolation since the suspension of classes	Total social isolation	0	0
			I am in social isolation, leaving only a few times a week for essential situations (e.g., grocery store, pharmacy, etc.)	Leave few times	1	1
			Whenever possible, I am in social isolation, but I need to leave to work	Leave to work	3	2
			I am not in social isolation	Not in social isolation	2	
		On average, how many hours per day have you been sleeping during the quarantine? (considering the 24-hour period)	Open-ended question	< 7h	0	0
				7h – 9h	1	1
				> 9h	2	2
		Has your body weight changed during the quarantine?	Decreased	Decreased	0	0
			Remained the same	Remained the same	1	1
			Increased	Increased	2	2
		In addition to attending classes, what type(s) of activity/activities have you participated in?	None	No	0	0
			Teaching activities	Yes	1	1
			Research activities			
			Extension activities			
			Others activities			

Abbreviations: IPAQ-SF = International Physical Activity Questionnaire - short form; NR = not reported; PA = physical activity; PAR = Physical Activity Rating; WHO: = World Health Organization. . * WHO PA recommendation (2020).
